# COVID-19 and the ethics of quarantine: a lesson from the Eyam plague

**DOI:** 10.1007/s11019-020-09971-2

**Published:** 2020-08-05

**Authors:** Giovanni Spitale

**Affiliations:** Institute of Biomedical Ethics and History of Medicine, University of Zurich, Winterthurerstrasse 30, 8006 Zurich, Switzerland

**Keywords:** History of medicine, History of epidemiology, Eyam, Plague, SARS-CoV-2, COVID-19, Public health ethics

## Abstract

The recent outbreak of the SARS-CoV-2 coronavirus is posing many different challenges to local communities, directly affected by the pandemic, and to the global community, trying to find how to respond to this threat in a larger scale. The history of the Eyam Plague, read in light of Ross Upshur’s Four Principles for the Justification of Public Health Intervention, and of the Siracusa Principles on the Limitation and Derogation Provisions in the International Covenant on Civil and Political Rights, could provide useful guidance in navigating the complex ethical issues that arise when quarantine measures need to be put in place.

## Introduction

The recent outbreak of the SARS-CoV-2 coronavirus is not an exclusively medical issue. The history of medicine and contemporary reflection clearly teach how an epidemic may have deep and sometimes radical social implications (Cohn 2002). After all, it is sufficient to keep an eye on the news of the day to recognise the fact: in addition to information on the progress of the disease, on the efforts of the scientific community to find a cure, or on the conditions of cities under quarantine, since the beginning of the outbreak newspapers from all over the West reported unfriendly, suspicious and sometimes openly racist attitudes towards people of Asian origin (Hussain 2020; Iqbal 2020; Lindrea and Gillett 2020; Ling 2020). The Twitter hashtag #JeNeSuisPasUnVirus, "I am not a virus" has become—the pun is not intentional, but hard to avoid—viral, used by thousands of users around the world to raise the level of public attention on the upsurge of xenophobia, "justified" (quotes are a must) by the fear of contagion. As the outbreak progresses and hits new countries, accompanied by its toll of panic, the same irrational dynamics could easily regard people with different origins. After the initial phase of virus entry into a new country, other divisions emerged, in this case not based on ethnicity but between different social groups, accompanied by the same load of suspicion and distrust. In the USA face masks have been resemantized from personal protective equipment to political symbols and statements, visually marking the division between “smug liberals” and “reckless republicans” (Lizza and Lippman 2020; Vetterkind 2020). In Italy, during the hardest phase of the lockdown, categories allowed to leave their houses, like dog owners, have been heavily stigmatized by so-called “balcony watchdogs”, and multiple sources have reported dogs killed by poisoned bites (BresciaToday 2020; Berton 2020; La Gazzetta del Mezzogiorno 2020). It seems that, together with the death toll and the incredible strain on health care systems, this pandemic brought us a steady corrosion of our societies’ social fabric. Although reactive institutions and social order are helping to avoid radical episodes, it is inevitable to note sociological affinities with the generalized and execrable suspicion towards entire human categories—Jewish people—that characterized many Black Plague outbreaks since 1348 (Finley and Koyama 2018). It is mandatory to point out that, in parallel with these divisive processes, many initiatives of diametrically opposite sign have punctuated lockdowns: togetherness has been expressed all over the world singing together from the balconies, applauding health care staff, volunteering for running errands for elders or other particularly vulnerable people, and so on. Nevertheless, social corrosion seems to be a stable companion of epidemics and quarantines, and as such an important side effect to consider, study and counteract.

This paper aims to offer two reflection standpoints for reflecting on whether and how it is possible to put in place ethically acceptable containment measures in the context of epidemics. One is historical, represented by the Eyam Plague, and one theoretical, offered by Upshur’s Four Principles for the Justification of Public Health Intervention and by the Siracusa Principles on the Limitation and Derogation Provisions in the International Covenant on Civil and Political Rights.

## Eyam plague

A few years ago I had the opportunity to visit the Peak District, in the UK. In an isolated Derbyshire valley I found the village of Eyam, sadly known to anyone with at least some familiarity with the history of epidemiology. In September 1665 the village was hit by a very serious plague epidemic, which decimated the small community: in October 1666, at the end of the epidemic, 257 of the approximately 700 people living in Eyam had died (Whittles and Didelot 2016).

**Fig. 1:**
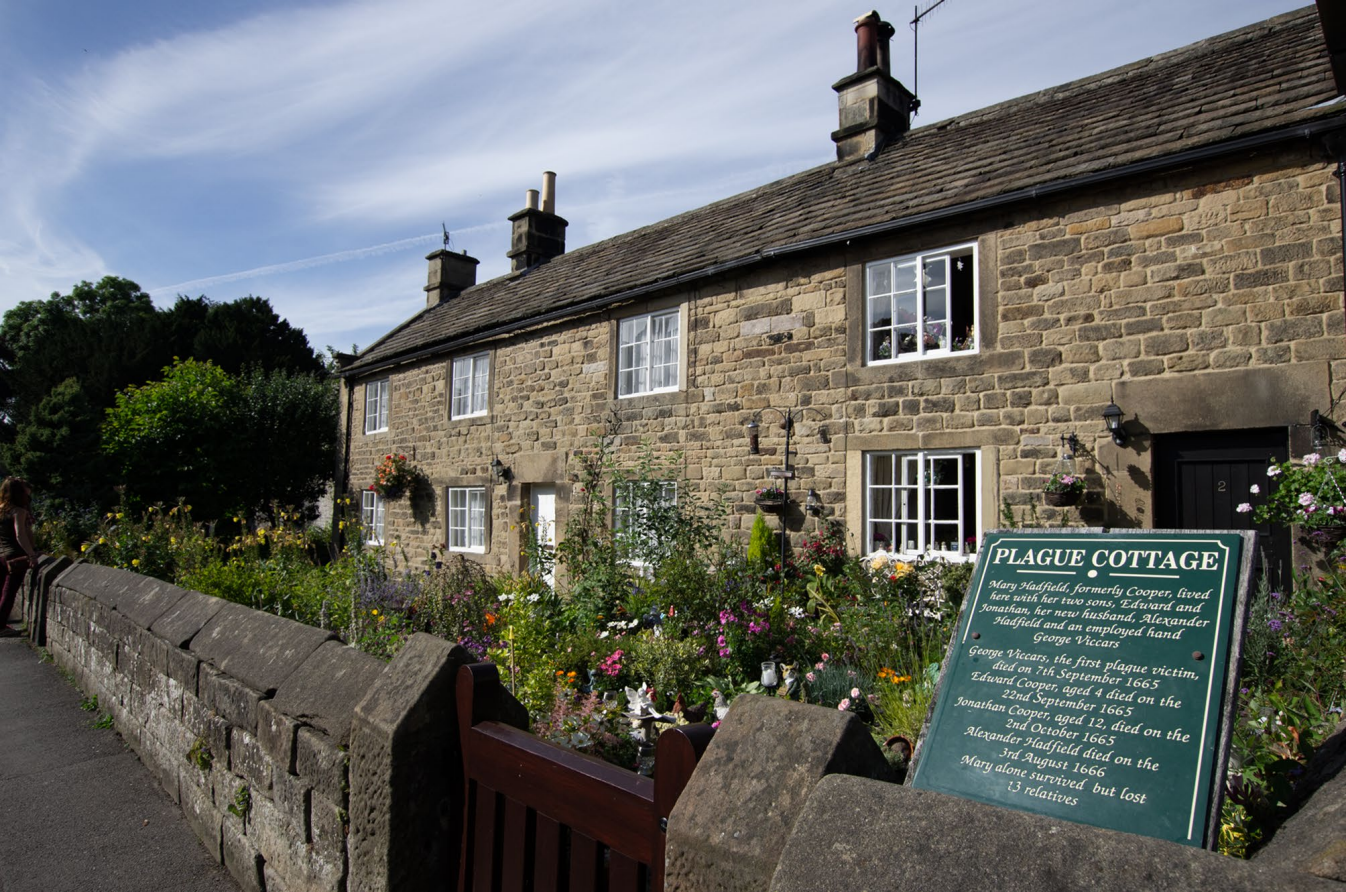
"Plague Cottage", former residency of the Hadfield family

According to the tradition, based on local chronicles (Wallis 2006) and stratified in various nineteenth-century literary narratives, the contagion was caused by a box of clothes imported from London by Alexander Hadfield, the village tailor. A few days after receiving the package, probably infested with infected fleas, George Viccars, Hadfield's assistant, died of the plague. Although some modern epidemiological studies accept this version (Massad et al. 2004), the real cause of the epidemic remains unclear: several authors believe, for example, that the outbreak of the epidemic was rather caused by an enzootic reservoir of wild rodents (Coleman 1986) (Fig. 1).

One point on which nineteenth-century chronicles and contemporary studies are in agreement is the management of the epidemic by the citizens of Eyam, at least peculiar for the time. Although the mechanics of the contagion were not clear, the first response to plague epidemics in the seventeenth century was often quarantine. This measure was detested by those who were subjected to it, and often violently opposed: in this sense, among the many, can be appreciated the contemporary testimonies of Samuel Pepys, an eyewitness to the Great Plague of London in 1665–1666 (Pepys 1893, vol. IV).

In London, Pepys writes, the limitation of contagion required drastic measures: “a watch is constantly kept there night and day to keep the people in, the plague making us cruel, as doggs[sic], one to another” (Pepys 1893). It is a sentence that deeply echoes Hobbes’ “homo homini lupus”, depicting a rapid and radical disruption of social fabric, strong enough to cast back London to that state of nature intended as “bellum omnium contra omnes”.

In Eyam, however, things took a different turn: the parish priest William Mompesson persuaded the local population about the need to establish a cordon sanitaire, placing the village in voluntary quarantine to protect other communities in the region from contagion (Wallis 2006).

The concept of “voluntary quarantine” is of particular interest: “quarantine”, from the Italian word “quaranta” was a sanitary measure introduced for the first time by the Most Serene Republic of Venice in 1377, during a plague outbreak in Dubrovnik and on the Dalmatian coast. Plague was spread by ships sailing from the eastern Mediterranean, and thus “if there was suspicion of disease on the ship, the captain was ordered to proceed to the quarantine station, where passengers and crew were isolated and the vessel was thoroughly fumigated and retained for 40 days” (Tognotti 2013). Historically, all over Europe quarantine was always imposed, and often enforced with firm measures. This is the reason why the case of Eyam is so peculiar: the quarantine was not imposed by an external authority, but the result of a persuasion process—and of a negotiation process—between William Mompesson and the local residents. As Sharp reports, “When the plague become worse, his wife besought him to leave the place, but he refused to do so. Moreover, he induced a number of the villagers, who wished to leave, to abandon their intention, by pointing out to them that they would carry the disease with them, and be a danger where ever they went. At the same time he wrote to the Earl of Devonshire, stating that the people would stay in Eyam if they were supplied with the necessaries of life” (Sharp 1898).

I remember two of the most interesting points during my visit to Eyam. The first: Cucklett Church, a "church without a church". Concerned that mass might contribute to spreading contagion, William Mompesson began saying mass outdoors, at this limestone platform (Sharp 1898). The second: Mompesson's well, an exchange point on the northern border of the county, used by residents of neighbouring towns to leave food and medicine to the quarantined community (Sharp 1898).

Some contemporary authors have hypothesized that in reality these measures may have contributed to increasing the mortality rate among the citizens of Eyam: according to Massad et al. “the hypothesis that confinement facilitated the spread of the infection by increasing the contact rate through direct transmission is plausible” (Massad et al. 2004); nevertheless, it remains clear how this “voluntary quarantine policy was humanitarian in intent; it was logically consistent with prevailing knowledge of plague, and it was pursued with great courage in the face of huge losses” (Coleman 1986).

The plague of Eyam ended in October 1666, leaving behind 257 deaths and a series of questions, some of them of a markedly ethical nature. What measures should be taken to try to limit an epidemic? What are justifiable, and if so, by what principles? Where to draw the line between the rights of individuals and the interest of communities? How to manage the different (and competing) interests of neighbouring communities?

## Disentangling causes and effects?

In order to understand what causes this corrosion of the social fabric that characterized most of the epidemic outbreaks (but not Eyam’s), it would be important to try untangling what can be imputed to the epidemic itself, and what to quarantine measures. In a recent review on the psychological impact of quarantine, Brooks et al. tried to summarize how this kind of measures impacts on people’s psychological health (Brooks et al. 2020). Considering recent epidemics and pandemics (2003–2019) they identified five stressors during quarantine (duration, fear of infection, frustration and boredom, lack of supplies, lack of information) and two post-quarantine stressors (finances and stigma).

Looking to this list it is immediately clear how deeply these issues are intertwined. “Fear of infection”, for instance, is clearly caused by an ongoing epidemic, even if quarantine measures can make people more aware of it and somehow hasten it. In this context we can definitely say that more research is needed, maybe comparing ethnographic studies conducted in places where quarantine measures were not imposed during the COVID-19 pandemic versus others conducted in quarantined areas.

What we know for sure is that epidemic outbreaks and quarantine measures to some degree contribute in creating a climate of fear, insecurity, and competition for scarce resources, resulting in the polarization of existing divisions. But, again, not in Eyam. Was it an idyllic village with no pre-existing differences that could be exacerbated by these phenomena? Not quite: as reported by Wallis, Mompesson had to craft and implement his plan together with Thomas Stanley, previous rector of Eyam (and still supported by many inhabitants) until his eviction for non-conformity, dating to 1662 (Wallis 2006). At least one, very deep social division based on religious credo was there, ready to blow. But it did not.

## Principles of quarantine ethics

To date, the reflection on public ethics and ethical response in the context of epidemics and pandemics revolves mainly around four approaches: deontological (or Kantian), utilitarian, principlist and casuistry (Coughlin 2006). Ross Upshur proposed an interesting epidemiological adaptation of the standard principles of Beauchamp and Childress, introducing a framework specifically designed for situations where quarantine measures are necessary:

1. Harm: the restriction of the freedoms of individuals or groups can only be justified if it is indispensable to avoid causing harm to others;
2. Least restrictive or coercive means: any action justified by the first principle should always use the mildest possible measures. In other words, education and discussion should precede prohibition or regulation;
3. Reciprocity: societies within which public health measures are taken must be prepared to compensate for any inconvenience caused to individuals or groups subject to such measures;
4. Transparency: all stakeholders affected by public health measures must be involved in the whole decision-making process, and the decision-making process must be as clear as possible (Upshur 2002, 2003).

The history of the Eyam pestilence proves to be a paradigmatic case, bearing in mind the limited medical knowledge available at the time, when read in the light of Upshur’s approach:

1. The limitation of the freedom of movement of the citizens of Eyam, through the establishment of the cordon sanitaire, was justified by the risk of spreading the contagion in the region;
2. The quarantine measures used were in fact concerted, relatively mild and accompanied by information on the prevention of contagion (such as outdoor masses);
3. The surrounding villages provided continuous material support to the population of Eyam;
4. In contrast to what happened in London—according to Pepys' diaries—quarantine decisions were not imposed in Eyam, but were rather discussed openly within the community.

The empirical test of theories in the field of public health ethics is often a problematic matter, if not a daunting task. But still it is needed, in order to assess the validity of a specific approach in managing complex situations in which decisions are critical and come with a price, often a heavy one. That is why the history of the Eyam plague is so valuable: because it gives some hints about how things could go, adopting a similar approach. Upshur’s principles could allow establishing a quarantine without having to impose it, in line with the suggestions of Brooks et al. in terms of mitigation strategies for quarantine’s psychological effects: keeping it as short as possible, providing adequate supplies, paying special attention to communication and quality information, reinforcing the altruistic effects (Brooks et al. 2020). Everything looks simple, on paper and retrospectively. It is not, especially when dealing with such a complex topic. Upshur’s principles are not so simple or straightforward to apply in a situation like the current one. First, and fundamental, drawing the line between individual rights and community interest is all but an easy task. One could argue that when an individual right (e.g. not having to bear the burden of a face mask) jeopardizes community interest (e.g. limiting the spread of an infectious disease) then it is fair to limit or suspend it. A straightforward libertarian would not accept such an argument, but should agree when considering “community interest” as an epiphenomenon resulting from the right to life and health of many other individuals. If this holds true for a trivial example as the “burden” of a face mask versus life and health, things become more tricky when confronting life and death of unknown others with the (potentially total) income loss due to social distancing, so the (potentially total) loss of livelihood to provide for one’s dear ones.

This is why, following the second principle, these measures need to be not only as mild as possible, but more properly as short as possible. Heavily uncertain scenarios demand flexibility, but people might be more willing to bear a stricter quarantine for a shorter period than a longer one, even if more relaxed (Brooks et al. 2020).

International solidarity risks to be hollowed to a bold claim with no substance, in a time in which Countries compete to be the first ones to secure themselves pre-emption rights on critical resources such as ventilators, face masks, drugs or vaccines (HHS 2020). Before embarking in such competitions, governments should seriously consider what kind of message they are giving, when on the one hand they ask their citizens to behave considerately and jointly, while on the other they act like the blindest utilitarian. This is something to take into account, when dealing with Upshur’s third principle, reciprocity, on a broader scale. In Upshur’s formulation, compensation is grounded on solidarity, and solidarity has nothing to deal with the aggressive international competition for scarce and critical resources mentioned above.

Upshur’s fourth principle—transparency—needs an important integration in order to be applicable in contemporary democracies bigger than a tiny English village of the seventeenth century, and this integration is offered by the Siracusa Principles on the Limitation and Derogation Provisions in the International Covenant on Civil and Political Rights: “every limitation of personal freedoms”, states the document, “should be discussed and applied by law, and not in an arbitrary manner”. As Brooks et al. note, we lack studies comparing the effects of voluntary versus enforced quarantine (Brooks et al. 2020). But it is legitimate to hypothesize that when a quarantine is perceived as the result of a discussion, either direct or by representatives, and when enough information is provided to stress how this could help keeping safe other members of a community, particularly the vulnerable ones, people could be more inclined to compliance in self-quarantining and suffer less adverse psychological outcomes.

Noncompliance will always be an issue. There will always be people that, even if properly informed, involved and compensated, will never accept even mild temporary measures. In a healthy democracy this is impossible to avoid. From a normative standpoint, the Siracusa Principles offer some guidance in whether and how it is justifiable to impose limitations to personal freedoms in order to protect and promote public health: Article 12 (freedom of movement), Article 18 (freedom of thought, conscience and religion), Article 19 (right to hold opinions), Article 21 (right of peaceful assembly) and Article 22 (freedom of association) include “protection of public health” as a reason for imposing limitations (The American Association for the International Commission of Jurists 1985).

In fact, during the 2005 outbreak of extensively drug-resistant tuberculosis in KwaZulu-Natal, South Africa (Singh et al. 2007), the WHO embraced this approach, stating that “if a patient wilfully refuses treatment and, as a result, is a danger to the public, the serious threat posed by XDR-TB means that limiting that individual's human rights may be necessary to protect the wider public. Therefore, interference with freedom of movement when instituting quarantine or isolation for a communicable disease such as MDR-TB and XDR-TB may be necessary for the public good, and could be considered legitimate under international human rights law” (WHO 2007), specifying that this approach must be considered a last resort. And a very sad one, one might argue.

As a side note, it is important to stress the fact that containing this pandemic and mitigating the transmission rate is a necessity, not only “just” to save human lives, but also in order to avoid much more critical situations in which much worse ethical issues arise. Italy already faced a hard time in this sense: the Italian Society of Anaesthesia Analgesia Reanimation and Intensive Care Therapy has recently released a document providing guidance on how to prevent, or at least postpone, the collapse of the health care system by changing the allocation criteria for ICU care. The document recommends to carefully assess, among other factors, age, severity of illness, comorbidities and life expectancy before deciding to admit patients to ICUs, because “It is not a question of making purely value choices, but of reserving resources that may be very scarce first for those who are more likely to survive, and secondly for those who may have more years of life saved, with a view to maximisation of the benefits for as many people as possible” (Vergano et al. 2020). Other countries followed shortly after in having to face this intense deliberation process (Joebges and Biller-Andorno 2020).

Allocation of scarce resources is a painful nut to crack, as widely discussed in the vast body of literature dealing with the topic (Dolan et al. 2005), and it is just an example of the kind of difficult ethical choices our societies will have to face, should we fail containing the pandemic. During a pandemic outbreak quarantine measures do need to be put in place as timely and efficiently as possible, and this needs to be done also ethically.

## Conclusion

Today's world is certainly more complicated than the rural English society of the 1600 s and the COVID-19 pandemic did not have its main outbreak in a village of 700 souls, but in Wuhan, a city of 11 million inhabitants, and that main outbreak has been followed by several others, scattered all around the world. It would be quite naive to infer that the same *actions* undertaken in Eyam could magically sort things out. Nevertheless, the history of Eyam and its voluntary quarantine, read in the light of Upshur’s *principles,* can be an interesting ethical paradigm, useful in providing guidance on how to understand and deal with some aspects of the current situation.

First of all, we must bear in mind that the people living today are not radically different from the people of the fourteenth or seventeenth century, and that our instinctive responses to frightening and incomprehensible phenomena such as epidemics tend to converge. For this reason it is imperative to provide not only timely, but also politically coordinated and unambiguous information and actions in order to reduce the margins where social chaos tends to develop, of which the current attitudes of suspicion and xenophobia are the clear prodromes.

Secondly, both at local level (i.e. where the pandemic has active clusters) and at global level, it is necessary to employ only measures that are justified by an actual risk, that—considering in the first place their safety and efficacy—are as mild and short as possible, and that are as concerted as possible with all relevant stakeholders.

Above all, the international community must recognize that principle of reciprocity formalized by Upshur and also acknowledged by WHO (WHO 2016, 30), providing continuous support—scientific, economic, logistical and human—to the communities affected by this pandemic. Recognizing the principle of reciprocity and writing policies based on it will not have the same symbolic power as bringing food to a place with romantic charm like the Mompesson well, but I do not see how this could reduce its validity.

A systematic reflection on these principles, before and while drafting measures meant to contain the pandemic, could help avoiding or at least mitigating that erosion of the social fabric and that radicalization of social conflicts that brought so much harm and that are again on the rise. We have a choice: to learn, reading the past in light of these reflections, or to constantly keep a watch, night and day, against the plague making us cruel as dogs one to another.
